# From Chaos to Clarity? The Quest for Effective Probiotics in Antibiotic-Associated Diarrhea

**DOI:** 10.1093/ofid/ofae616

**Published:** 2024-10-21

**Authors:** Krishna Rao, Kevin W Garey

**Affiliations:** University of Michigan Medical School, Ann Arbor, Michigan, USA; College of Pharmacy, University of Houston, Houston, Texas, USA

**Keywords:** antibiotic-associated diarrhea, *Clostridioides difficile*, probiotics, live biopharmaceuticals, randomized controlled trial

## Abstract

Antibiotic-associated diarrhea (AAD) frequently complicates treatment of infections. A recent randomized, double-blind, placebo-controlled trial tested a proprietary probiotic mixture and found that it reduced the incidence of AAD by 16%. This is encouraging for patients, but future progress on probiotics for AAD and other conditions depends on transparency around strain selection, probiotic design guided by preclinical mechanistic studies, and rigorously conducted human studies.

Antibiotic-associated diarrhea (AAD) remains a prevalent complication of antibiotic therapy, affecting a significant portion of patients and leading to decreased quality of life and increased health care costs [1]. Traditional treatments have limitations, and *Clostridioides difficile* infection and/or asymptomatic carriage can complicate the clinical picture [2]. Certain antibiotics have direct pharmacologic effects on the gastrointestinal tract causing diarrhea, including motilin-receptor agonists (erythromycin) and stimulation of small bowel motility (clavulanate) [3]. However, reduction of anaerobic gut bacteria is a common side effect of many antibiotics; this decreases the metabolism of nondigestible carbohydrates, stimulating osmotic diarrhea, and increases primary bile acids, which are potent colonic secretory agents [4, 5]. Since the microbiome is implicated in the pathogenesis of AAD [6], probiotics have been proposed as a potential solution, but their effects are highly specific to strain and disease [7]. The current body of probiotic research is fragmented, with numerous small studies varying widely in strains used, populations studied, and outcomes measured [8]. This heterogeneity has made it difficult to draw meaningful conclusions or develop clear clinical guidelines on probiotic studies to prevent or treat AAD.

A recent randomized, double-blind, placebo-controlled phase 4 trial evaluated the efficacy of a proprietary probiotic mix, Sinquanon, in reducing AAD [9]. The study included 564 participants, with 555 completing the trial. Participants received the probiotic mix during antibiotic treatment and for 14 days afterward. The results showed a significant ∼16% reduction in the incidence of AAD (number needed to treat, 7), with reductions in the severity and duration of diarrhea and improvements in quality-of-life measures. Participants who did experience AAD while taking Sinquanon had a 1-day reduction of diarrhea on average as compared with placebo. There were no significant differences in adverse events between the probiotic and placebo groups. The study was methodologically rigorous, featuring off-site randomization, coded medication packs, and instructions to avoid consuming other probiotics or yogurt.

While this positive outcome from a well-conducted study is promising, it raise questions about the generalizability and applicability of the results. The impressive outcome may stem from Sinquanon being a rationally designed probiotic with experimentally guided strain and nutrient selection, as it was deliberatly developed from first principles for the treatment of AAD, rather than being an extant, off-the-shelf product. However, the proprietary nature of the strains used limits our understanding of the specific mechanisms of action and the reasons behind each strain's inclusion. Without transparency regarding the genomes and benefitical functions of these strains, it will be difficult to take these insights and translate them to future probiotic therapies.

Moreover, the study did not conduct *C difficile* testing, leaving a gap in understanding the probiotic's impact on this significant pathogen often accompanying AAD, and the probiotic data for *C difficile* prevention are similarly heterogeneous and/or of poor quality [8]. Although participants were instructed to avoid fermented foods and other supplements, dietary information was lacking, which could be a confounding factor. Safety considerations remain, especially for patients who are immunocompromised, given reports of bacteremia associated with probiotic use [10–12]. The broader field of probiotic research suffers from this kind of opacity and heterogeneity. Meta-analyses often attempt to synthesize data from disparate studies, but the extreme underlying differences render such efforts akin to assembling a puzzle with mismatched pieces. Consequently, clinicians are left without clear guidance on which probiotics to recommend for specific conditions and for specific patient populations.

This study demonstrates that the development of probiotics for AAD is a tractable problem and encourages efforts to develop probiotics for other conditions. To move forward as a scientific community, there is a need for rigorously conducted studies such as this, using defined microbial consortia but with known genomes and mechanisms of action. Transparency and a focus on strain and nutrient selection, guided by a mechanistic understanding of the disease, will enable development of targeted probiotics tailored to specific diseases and patient populations. Such an approach would help piece together the fragmented landscape of probiotic research.

In conclusion, the Sinquanon study offers encouraging results for the use of probiotics in AAD. It also underscores the importance of (1) selecting strains and other important nutrients with guidance from mechanistic studies and (2) conducting rigorous, high quality human trials of probiotics. The field must embrace a rational, science-based probiotic design approach and open scientific collaboration to truly advance our understanding. Only through such efforts can we develop effective, evidence-based therapies that clinicians can confidently recommend, ultimately improving patient care in AAD and beyond.

## Notes

***Financial support*.** This work was supported in part by a grant from the Agency for Healthcare Quality and Research (R01-HS-027431 to K. R.); and a grant from the National Institute of Allergy and Infectious Diseases (NIH-NIAID R01 2R01AI139261 to K. W. G.).
